# Supplementary material for: Detection of PCR chimeras in adaptive immune receptor repertoire sequencing using hidden Markov models
Source: bioRxiv. 2025 Feb 26:2025.02.21.638809. Preprint. [Version 1] doi: 10.1101/2025.02.21.638809 (PMC11888211; doi:10.1101/2025.02.21.638809)
Supplement: Supplement 2 [file NIHPP2025.02.21.638809v1-supplement-2.pdf]

bioRxiv preprint doi: https://doi.org/10.1101/2025.02.21.638809; this version posted February 26, 2025. The copyright holder for this preprint (which was not certified by peer review) is the author/funder, who has granted bioRxiv a license to display the preprint in perpetuity. It is made available under aCC-BY 4.0 International license.

Supplementary figures

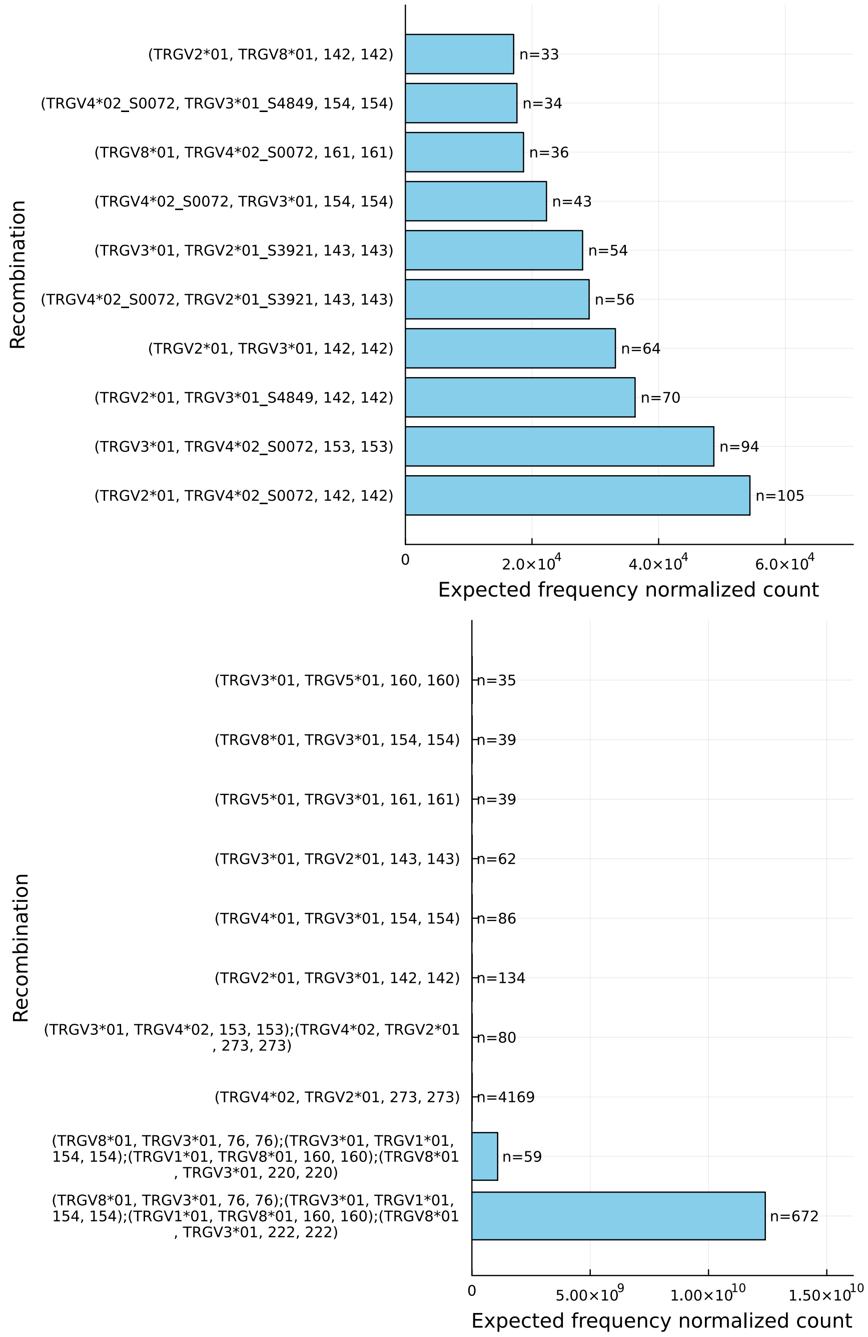

Supplementary Figure 1: The bar plots depict the normalized counts of recombinations found by CHMMAIRRa in the TRG/D04 library from the Corcoran et al. dataset analyzed with the (A) the IMGT database and (B) the donor's personalized genotype. Normalization was performed by dividing the occurrences of a recombination by the product of template allele frequencies. The overrepresented recombination in (B) is actually the TRGV4*02_S0072 allele, missing from the IMGT database.

bioRxiv preprint doi: https://doi.org/10.1101/2025.02.21.638809; this version posted February 26, 2025. The copyright holder for this preprint (which was not certified by peer review) is the author/funder, who has granted bioRxiv a license to display the preprint in perpetuity. It is made available under aCC-BY 4.0 International license.

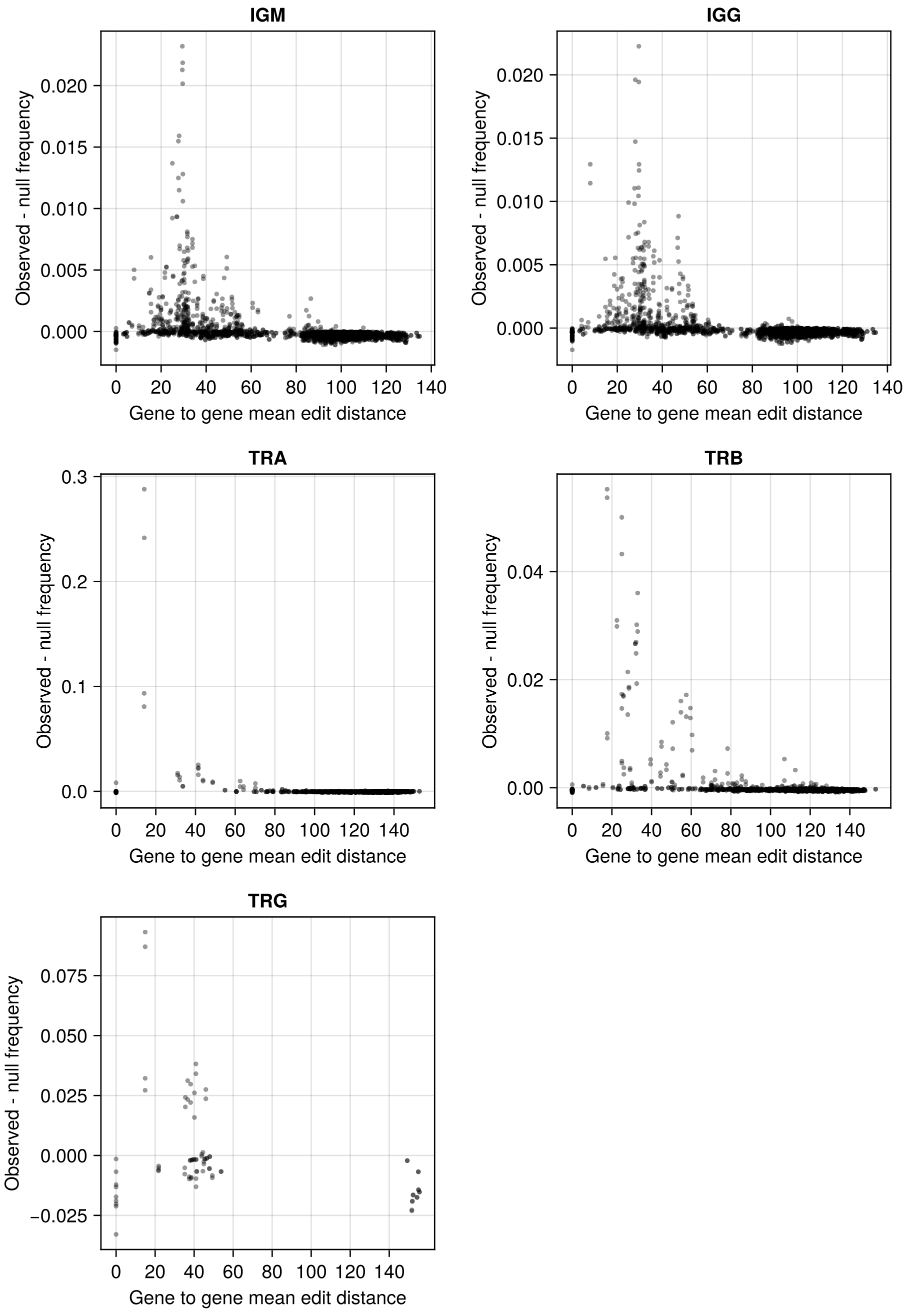

Supplementary Figure 2: Scatterplots demonstrating the relationship between gene to gene edit distance and overrepresented gene to gene recombinations. Each dot represents all recombinations involving two specific genes. The observed frequency refers to co-occurrence of genes in recombinations, averaged across all datasets utilized in this paper except the PCR conditions test dataset. The expected frequency was calculated by taking the product of the two genes' frequencies. Dots above y = 0 are recombinations which are observed more than expected by taking the product of the two genes' frequencies, while dots below y = 0 are observed less often than expected. Methods settings listed in Supplementary data 1 and datasets in the data availability section.

bioRxiv preprint doi: https://doi.org/10.1101/2025.02.21.638809; this version posted February 26, 2025. The copyright holder for this preprint (which was not certified by peer review) is the author/funder, who has granted bioRxiv a license to display the preprint in perpetuity. It is made available under aCC-BY 4.0 International license.

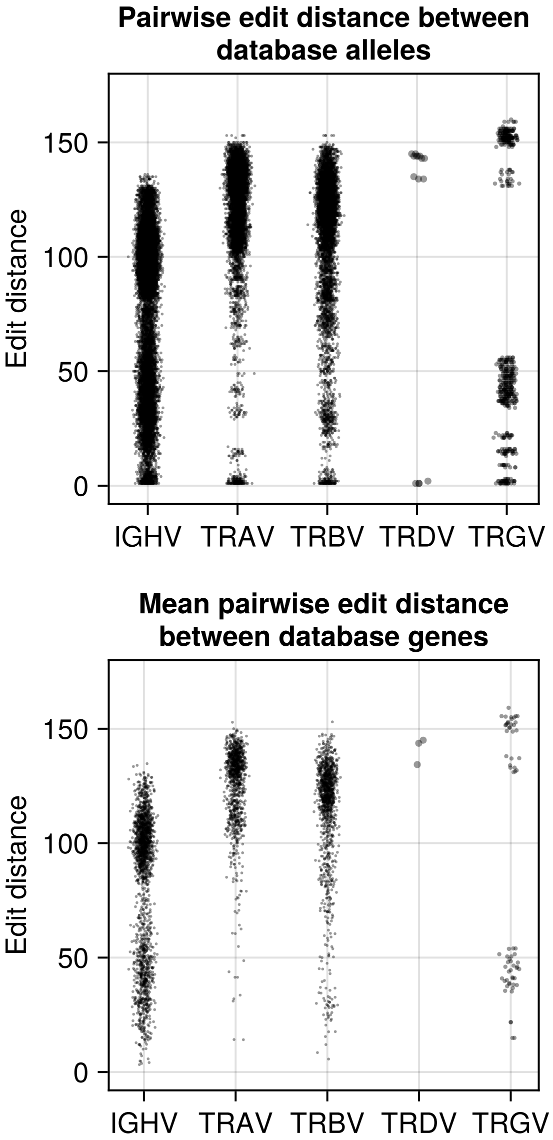

Supplementary Figure 3: Scatterplots depicting the distribution of Levenshtein distances (A) between all pairs of V alleles and (B) averaged per-gene. The lack of similar TRDV alleles may be related to the lack of chimeras in TRD libraries. The OGRDB release 9 was used for the IGHVs and the KI TCR database v0.0.1 was used for the TCRVs. Database versions listed in Supplementary data 1.
